# Key Role of the GITR/GITRLigand Pathway in the Development of Murine Autoimmune Diabetes: A Potential Therapeutic Target

**DOI:** 10.1371/journal.pone.0007848

**Published:** 2009-11-20

**Authors:** Sylvaine You, Lynn Poulton, Steve Cobbold, Chih-Pin Liu, Michael Rosenzweig, Douglas Ringler, Wen-Hui Lee, Berta Segovia, Jean-François Bach, Herman Waldmann, Lucienne Chatenoud

**Affiliations:** 1 Université Paris Descartes, Paris, France; 2 INSERM, Unité 580, Paris, France; 3 University of Oxford, Sir William Dunn School of Pathology, Oxford, United Kingdom; 4 Department of Immunology, Department of Diabetes, Endocrinology and Metabolism, Beckman Research Institute, City of Hope, Duarte, California, United States of America; 5 Tolerx, Inc., Cambridge, Massachusetts, United States of America; New York University School of Medicine, United States of America

## Abstract

**Background:**

The cross-talk between pathogenic T lymphocytes and regulatory T cells (Tregs) plays a major role in the progression of autoimmune diseases. Our objective is to identify molecules and/or pathways involved in this interaction and representing potential targets for innovative therapies. Glucocorticoid-induced tumor necrosis factor receptor (GITR) and its ligand are key players in the T effector/Treg interaction. GITR is expressed at low levels on resting T cells and is significantly up-regulated upon activation. Constitutive high expression of GITR is detected only on Tregs. GITR interacts with its ligand mainly expressed on antigen presenting cells and endothelial cells. It has been suggested that GITR triggering activates effector T lymphocytes while inhibiting Tregs thus contributing to the amplification of immune responses. In this study, we examined the role of GITR/GITRLigand interaction in the progression of autoimmune diabetes.

**Methods and Findings:**

Treatment of 10-day-old non-obese diabetic (NOD) mice, which spontaneously develop diabetes, with an agonistic GITR-specific antibody induced a significant acceleration of disease onset (80% at 12 weeks of age). This activity was not due to a decline in the numbers or functional capacity of CD4^+^CD25^+^Foxp3^+^ Tregs but rather to a major activation of ‘diabetogenic’ T cells. This conclusion was supported by results showing that anti-GITR antibody exacerbates diabetes also in CD28^−/−^ NOD mice, which lack Tregs. In addition, treatment of NOD mice, infused with the diabetogenic CD4^+^BDC2.5 T cell clone, with GITR-specific antibody substantially increased their migration, proliferation and activation within the pancreatic islets and draining lymph nodes. As a mirror image, blockade of the GITR/GITRLigand pathway using a neutralizing GITRLigand-specific antibody significantly protected from diabetes even at late stages of disease progression. Experiments using the BDC2.5 T cell transfer model suggested that the GITRLigand antibody acted by limiting the homing and proliferation of pathogenic T cells in pancreatic lymph nodes.

**Conclusion:**

GITR triggering plays an important costimulatory role on diabetogenic T cells contributing to the development of autoimmune responses. Therefore, blockade of the GITR/GITRLigand pathway appears as a novel promising clinically oriented strategy as GITRLigand-specific antibody applied at an advanced stage of disease progression can prevent overt diabetes.

## Introduction

The glucocorticoid-induced tumor necrosis factor receptor (GITR, also known as TNFRSF18) belongs to the TNF-nerve growth factor receptor gene superfamily and is expressed by a variety of immune cells. Resting CD4^+^ and CD8^+^ T cells, NK cells, B cells, macrophages and dendritic cells (DCs) express low levels of GITR [1]. At the T cell surface, GITR expression increases following activation. Attention was initially drawn to GITR as a new marker for CD4^+^CD25^+^ regulatory T cells (Tregs), essential for the control of a variety of immune responses (autoantigens, infectious and tumor antigens, allergens and alloantigens) [2], [3] which constitutively express high levels of the molecule [1], [4]. Several reports suggest that signaling through GITR abrogates the suppressive functions of Tregs. *In vitro*, addition of mouse-specific anti-GITR antibodies reverses the *in vitro* suppressive capacity of Tregs [1], [4]. It has been demonstrated that GITR acts as a costimulatory molecule [5], [6]. Thus, GITR triggering enhances T cell proliferation and cytokine production in response to T cell receptor (TCR) stimulation. Moreover, GITR cross-linking inhibits T cell receptor-induced apoptosis [7]–[9] and sustains T cell survival and responsiveness by triggering three distinct MAPKs pathways (ERKs, JNKs and p38) and activating NF-κB [6], [10], [11].

In the mouse, the ligand of GITR (GITRL) is expressed on endothelial cells and antigen presenting cells (APCs) including dendritic cells (DCs), macrophages and B cells [5], [6], [12]. Its expression is up-modulated by various pro-inflammatory stimuli [5], [6]. At variance, in the human a more restricted distribution was described. Besides endothelial cells, human GITRL is exclusively detected on activated plasmacytoid DCs (but not on T cells, B cells, NK cells, macrophages, mature or immature myeloid DCs) [13], [14]. In addition, over expression of GITRL in human monocyte-derived DCs enhances their capacity to activate T cells by providing costimulatory signals [14]. In macrophages, GITRL signaling leads to the production of pro-inflammatory mediators such as IL-1β, IL-8, TNF-α, MCP-1, inducible nitric oxide synthetase (iNOS) and matrix metalloproteinase (MMP)-9 [15], [16]. Interestingly, a recent report showed that, in mouse, plasmacytoid DCs reverse signaling through GITRL could also activate indoleamine 2,3-dioxygenase (IDO) thus inducing a major down-regulatory loop though the tryptophan catabolism regulatory pathway [17].

Compelling evidence has accumulated to show that *in vivo* triggering of GITR with an agonistic monoclonal antibody significantly up-regulates immune responses to tumors and infectious agents thus facilitating their eradication, and so representing a suitable ‘adjuvant’ strategy in these situations [18]–[22]. In keeping with these observations are data showing that treatment with anti-GITR also exacerbates the development of autoimmune and allergic disorders including autoimmune gastritis [1], experimental autoimmune encephalomyelitis (EAE) [23], experimental collagen-induced arthritis (CIA) and asthma [24]. As a mirror image of these findings, GITR-deficient mice (GITR^−/−^) show significantly reduced inflammatory reactions as compared to wild-type animals in response to various stimuli i.e. chronic lung injury induced by bleomycin instillation, type II collagen-induced arthritis, TNBS (2,4,6-trinitrobenzene sulphonic acid)-induced colitis [25]–[27]. *In toto*, these data strongly suggest that, if properly manipulated, the GITR/GITRL pathway could represent an interesting therapeutic target in quite distinct settings. As mentioned, GITR triggering is beneficial in the infectious and tumor setting. Conversely, in the particular case of autoimmunity, while GITR activation appears counterproductive, no reagents were available until recently to assess whether effective blockade of the GITR/GITRL interaction could inhibit lymphocyte autoreactivity and prevent autoimmunity.

In this study, we have examined, for the first time, the role of the GITR/GITRL pathway in the course of a spontaneous autoimmune disease, insulin-dependent type 1 diabetes, using the non-obese diabetic (NOD) mouse that in many aspects recapitulates the human disease [28]. Our data demonstrate, in keeping with previous reports in other autoimmune conditions, that GITR triggering exacerbates autoimmune diabetes. However, our results extend previous findings in showing that this accelerating effect is a consequence of selective activation/costimulation of pathogenic T cells while Tregs are spared. A second important and novel finding is that a blocking antibody to GITRL can effectively prevent the onset of diabetes.

## Materials and Methods

### Mice

NOD (K^d^, I-A^g7^, D^b^), BDC2.5 NOD, CD28^−/−^ NOD (kindly provided by J. Bluestone, Diabetes Center and Department of Medicine, University of California, San Francisco) and NOD-SCID mice were bred in our animal facilities under specific pathogen-free conditions. Colorimetric strips were used to monitor glycosuria (Glukotest, Boehringer-Mannheim, Mannheim, Germany) and fasting glycemia (Haemoglukotest and Reflolux F; Boehringer-Mannheim).

Ethical statement: All experiments have been conducted in accordance with European Union Council Directives (86/609/EEC) and with institutional guidelines (INSERM: Institut National de la Santé et de la Recherche Médicale). The animal facility has an agreement delivered by the *Prefecture de Police* of Paris, France.

### Antibody Treatment

Ten day-old NOD or CD28^−/−^ NOD mice were treated with anti-GITR antibody (rat IgG2a antibody 2F8) (Tolerx Inc., Cambridge, MA) or purified mouse IgGs (Jackson laboratories, West Grove, PA). The dose used was 0.2, 0.4 or 0.8 mg/injection i.p. on d10, d17 and d24 of life. Anti-GITRLigand antibody (rat IgG1 antibody YGL 386.2 [5]) was administered i.p. at the dose of 1 mg/injection. Eleven week-old NOD mice received 1 mg/week for 4 weeks. In another experiment, anti-GITRL treatment was started at 6 weeks of age in CD28^−/−^ NOD mice (week 6 to 9 included).

### Histology

Stomach, pancreas, salivary glands and thyroid were fixed in 4% formalin and processed according to standard methods. Five µm thick paraffin sections stained with hematoxylin-eosin-safran were examined.

### Adoptive Transfer

Total splenocytes were recovered from 32-week-old diabetes-free NOD mice that have been treated with anti-GITRL antibody (from week 11 to 14 of life). After depletion of B cells by magnetic bead cell sorting (Miltenyi Biotech), Tregs were removed on the basis of their expression of CD25 or CD62L. Purity of the sorted cells was 90–95%. CD25^−^CD62L^−^ T cells were then injected i.v. into 6-week-old NOD-SCID mice (10^6^/recipient) and diabetes was monitored 2 times a week until disease occurred.

### Infusion of CFSE-Labeled BDC2.5 Cells

CD4^+^ T cells were purified from BDC2.5 NOD splenocytes [29] by magnetic cell sorting (Miltenyi Biotech, Germany). BDC2.5 T cells represent more than 80% of the CD4^+^ population [30]. The cells were labeled with carboxyfluorescein diacetate succinimidyl ester (CFSE, 1 µM, 10 min at 37°C) and transferred i.v. into 3 to 4-wk-old NOD mice (10^7^/recipient). Recipient animals were treated either with anti-GITR (1 mg/injection) or with anti-GITRLigand antibodies (1 mg/injection) on day 0, 1 and 4 after BDC2.5 T cell infusion. Pancreas, spleen, pancreatic and mesenteric lymph nodes were harvested on day 7. Pancreatic islets were isolated by Histopaque gradient (Sigma, France) after collagenase P digestion (0.6 mg/ml, 15 min at 37°C) and infiltrating T cells were subsequently collected after islet trypsin/EDTA treatment.

### Flow Cytometry

CD25, CD4, CD8, CD44 and CD69 antibodies were obtained from PharMingen-BD (San Diego, CA). The biotinylated anti-GITR antibody (clone YGITR 765.4, rat IgG2b) previously described [5], [31] was used. Foxp3 intracellular staining was performed according to the manufacturer's instructions (eBioscience, San Diego, CA). The class II MHC tetramer carrying a BDC2.5 T cell-specific mimotope (tetAg7/p79) was used as previously described [30]. Briefly, cells were stained with PE-labeled tetramers (5 µg/ml) at 37°C for 3 hrs. Cell surface antibodies were added during the last 30 min of incubation.

### *In Vitro* Proliferation Assays

CD4^+^CD25^+^ and CD4^+^CD25^−^ T cells (2×10^4^ cells/well) were cultured at a 1∶1 ratio and stimulated with CD3 antibody (2.5 µg/ml) and APCs. To assess GITR costimulatory function, CD4^+^CD25^+^ or CD4^+^CD25^−^ T cells were stimulated with CD3 antibody (145 2C11, 0.5 µg/ml) and increasing concentrations of anti-GITR antibody. In other experiments, CD4^+^CD25^+^ T cells were first stimulated with CD3 antibody alone (0.5 µg/ml) or in combination with anti-GITR antibody (50 µg/ml) for 48 hrs and were then co-cultured with freshly isolated CD4^+^CD25^−^ T cells in the presence of APCs and CD3 antibody. In all settings, cells were incubated for 72 hrs and pulsed with [^3^H]-thymidine (Amersham). Data from the co-cultures were expressed as the % inhibition = [1−(cpm (CD4^+^CD25^−^ plus CD4^+^CD25^+^)/cpm CD4^+^CD25^−^)]×100.

### Elispot Assay

PVDF plates (Millipore, St Quentin-en-Yvelines, France) were coated overnight with anti-IFN-γ antibody (U-Cytech, Utrecht, the Netherlands). CD4^+^CD25^−^ or CD8^+^ T cells were added (1.5×10^5^/well) and stimulated with CD3 antibody (0.05 µg/ml) and increasing concentrations of anti-GITR antibody for 20 hr. In additional experiments, CD4^+^BDC2.5 T cells were cultured with an agonist mimotope peptide (1040-51, 2 ng/ml). After cell removal, IFN-γ secretion was detected with biotinylated anti-IFN-γ antibody, streptavidin-horseradish peroxidase and 3-amino-9-ethylcarazole (AEC). All IFN-γ spot readouts were expressed as spot-forming cells (SFC)/10^6^ cells.

### Statistical Analysis

The occurrence of diabetes was plotted using the Kaplan-Meier method. The statistical comparison between the curves was performed using the logrank (Mantel-Cox) test. In addition, results were analyzed using the Student's t test when appropriate.

## Results

### 1) GITR Triggering Boosts Diabetogenic T Cells in NOD Mice both *In Vivo* and *In Vitro*

As already mentioned, in various models of autoimmunity (autoimmune gastritis, EAE, CIA), disease exacerbation has been reported following administration of an agonistic anti-GITR antibody, DTA-1, produced by the group of S. Sakaguchi [1].

Here, using another agonistic antibody, we studied the effect of GITR triggering in autoimmune insulin-dependent diabetes. To that aim, we used two different models, namely conventional NOD mice and BDC2.5 NOD mice that express a transgenic TCR derived from a pathogenic (‘diabetogenic’) CD4^+^ T cell clone [29].

#### a) Acceleration of insulitis and diabetes progression

**Figure 1:**
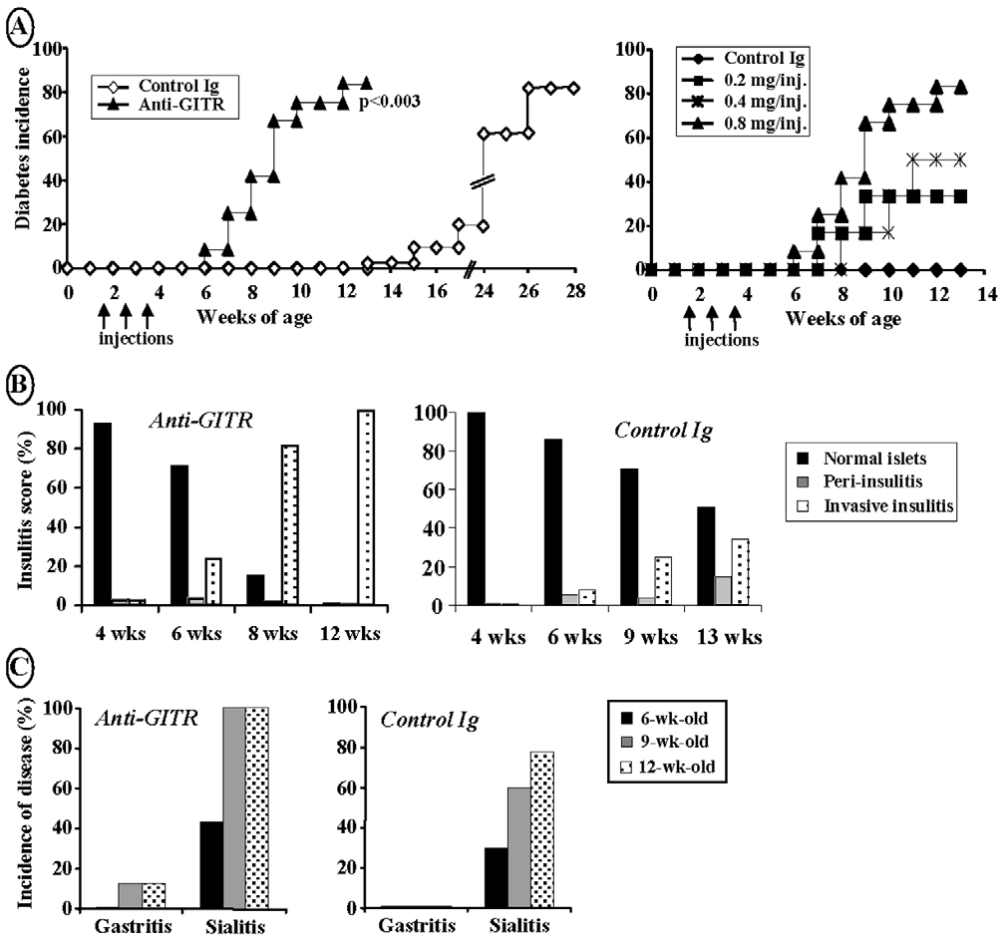
Exacerbation of diabetes incidence in NOD mice following treatment with anti-GITR antibody. (A) Ten day-old female NOD mice were treated with an antibody against GITR. Mice having received purified mouse IgGs were used as controls. The dose used was 0.2 mg (n = 6), 0.4 mg (n = 6) or 0.8 mg (n = 12)/injection/mouse i.p. once a week on d10, d17 and d24 of life. Onset of diabetes was significantly accelerated in a dose-dependent manner (for 0.8 mg/injection, p<0.003). (B) Histological analysis of pancreas from anti-GITR antibody-treated NOD mice killed at various ages. The severity of aggressive insulitis was significantly increased at 9 and 12 weeks of age (p<0.0005 of both ages). (C) Histological analysis of stomach and salivary glands of NOD mice treated with anti-GITR antibody.

Ten day-old female NOD mice were injected i.p. with increasing doses of a GITR-specific antibody, 2F8, (0.2, 0.4 or 0.8 mg/injection on day 10, 17 and 24 of life). The anti-GITR antibody used in our study, like the DTA-1 produced by the group of Sakaguchi [1], is agonistic. Control mice were treated with purified mouse IgGs. As shown in figure 1A, anti-GITR-treated mice showed a significant and quite impressive acceleration of overt diabetes onset; mice receiving the higher anti-GITR dose developed hyperglycemia and glycosuria by 6–8 weeks of age that is 5–7 weeks before disease onset in control NOD females. The effect was dose-dependent; at 12 weeks of age, diabetes was observed in 80%, 50% and 35% of NOD females treated with 0.8 mg, 0.4 mg or 0.2 mg of anti-GITR, respectively (figure 1A). Consistent with this marked acceleration of disease, histological examination of pancreata showed a significantly more rapid progression from benign to aggressive/destructive insulitis in anti-GITR-treated NOD mice as compared to controls (figure 1B). Thus, by 8 weeks of age, 82% of the islets were massively infiltrated as compared to 25% in control animals (figure 1B).

Anti-GITR-treated NOD mice were also examined for the occurrence of other autoimmune manifestations including sialitis, gastritis and thyroiditis (figure 1C). As compared to control mice, anti-GITR-treated NOD females exhibited more severe and rapidly progressing sialitis. Gastritis was also observed in 12% of the treated animals but remained modest. Finally, histological analysis did not reveal any sign of autoimmune thyroiditis (data not shown).

**Figure 2:**
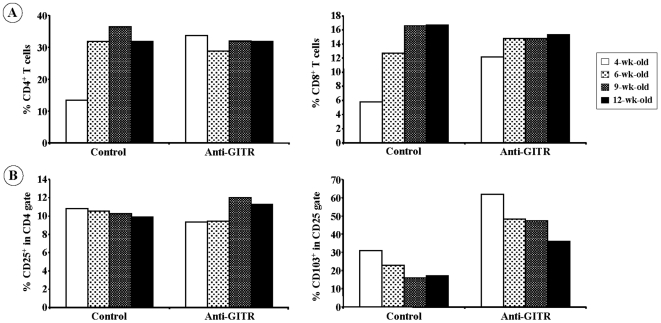
Phenotypic analysis of the splenic T cell compartment in anti-GITR antibody-treated NOD mice. (A) CD4 and CD8 staining in total spleen cells recovered from NOD mice treated with 0.8 mg/injection of anti-GITR antibody and sacrificed at different ages. (B) CD25 staining in CD4 gate and CD103 staining in CD4^+^CD25^+^ T cell gate.

Major lymphocyte subsets were analyzed in the spleen of anti-GITR-treated and control animals. At 4 weeks of age (i.e. 2–3 days after the last injection), anti-GITR-treated mice exhibited significantly higher proportions of both CD4^+^ and CD8^+^ T cells as compared to IgG-treated controls (33.7% versus 13.4% for CD4 and 12.2% versus 5.8% for CD8) (figure 2A). The pattern was back to normal by 2 weeks after the end of treatment (figure 2A).

At early time points after anti-GITR treatment, clear phenotypic evidence of T cell activation was observed within the CD4^+^ compartment as assessed by an increase in CD69 and CD44 expressing cells and a decrease in CD62L^high^ expressing cells (data not shown). Moreover, in anti-GITR-treated NOD mice, very high proportions of CD4^+^CD25^+^ T cells expressed CD103 (>60% at 4 weeks of age) (figure 2B), an effect that progressively declined after the end of the treatment (45% and 35% of CD103^+^ were observed at 9 or 12 weeks of age, respectively).

As already reported in the literature, GITR is constitutively expressed by CD4^+^CD25^+^ Tregs. The proportion of CD4^+^CD25^+^ T cells was not affected by anti-GITR antibody treatment. In treated animals, GITR-expressing cells were coated by the *in vivo*-injected antibody, a phenomenon which, in mice receiving the highest dose, lasted 4–5 weeks after treatment (data not shown).

#### b) GITR ligation on effector T cells is costimulatory *in vitro*

**Figure 3:**
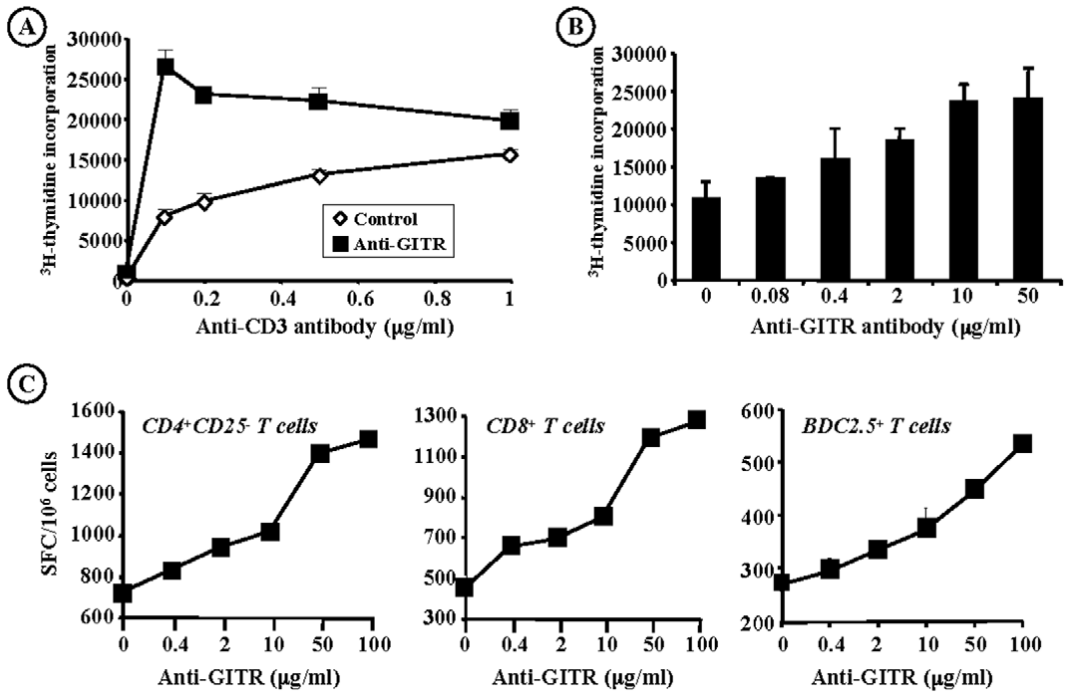
GITR acts as a costimulatory molecule on T cells. (A) Proliferation of CD4^+^CD25^−^ T cells issued from the spleen of 6-wk-old NOD mice injected with anti-GITR antibody (0.8 mg) or PBS (controls) on d10, d14 and d24 in response to increasing concentrations of anti-CD3 antibody. (B) CD4^+^CD25^−^ T cells recovered from the spleen of unmanipulated NOD mice were stimulated with coated CD3 antibody (0.5 µg/ml) in the presence of increasing concentrations of anti-GITR antibody ranging from 0.08 to 50 µg/ml. (C) IFN-γ Elispot assay. CD4^+^CD25^−^ or CD8^+^ T cells from unmanipulated NOD mice were stimulated (1.5×10^5^/well) with soluble CD3 antibody (0.05 µg/ml) and increasing concentrations of anti-GITR antibody (0.08 to 100 µg/ml) for 20 hrs. In the right panel, CD4^+^BDC2.5 T cells were used and stimulated with an agonist mimotope peptide (1040-51, 2 ng/ml) and anti-GITR antibody. IFN-γ spot readouts are expressed as spot-forming cells (SFC)/10^6^ cells. The results shown are representative of at least three independent experiments.

To further investigate the costimulatory role of GITR on pathogenic/effector T cells, CD4^+^CD25^−^ T cells from anti-GITR-treated or control NOD mice were purified and stimulated *in vitro* with increasing doses of CD3 antibody. Results showed that CD4^+^CD25^−^ T cells from anti-GITR antibody-treated NOD mice proliferated better even at the lowest dose of anti-CD3 (0.05 µg/ml: 29618 cpm as compared to 5050 cpm for controls; p<0.0001) thus confirming the increased activation pattern exhibited by CD4^+^ T cells following GITR triggering (figure 3A).

In another set of experiments, CD4^+^CD25^−^ T cells from non manipulated NOD mice cultured in presence of low concentrations of anti-CD3 showed a dose-dependent proliferative response following addition of increasing amounts of anti-GITR antibody (figure 3B). Moreover, using an ELISPOT assay, we measured IFN-γ production by CD4^+^CD25^−^ or CD8^+^ T cells after a 20 hr stimulation in presence of sub-optimal concentrations of anti-CD3 and increasing doses of anti-GITR (figure 3C). The same experiment was performed using transgenic CD4^+^BDC2.5 T cells stimulated with an agonist mimotope peptide (1040-51) (instead of anti-CD3). In both cases, addition of anti-GITR antibody resulted in a greater IFN-γ production in a dose-dependent fashion (figure 3C).

#### c) GITR triggering enhanced *in vivo* proliferation and migration of effector T cells to the pancreas

**Figure 4:**
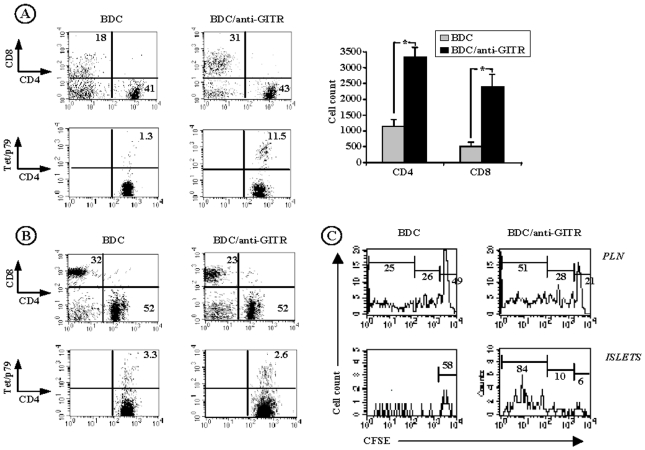
*In vivo* GITR costimulatory action on BDC2.5 T cells. Three to four-week-old NOD mice were infused with 10^7^ CFSE-labeled CD4^+^BDC2.5 T cells and were treated or not with anti-GITR antibody on day 0, 1 and day 4 after transfer. Recipient mice were sacrified on day 7 and cell suspensions were recovered from the pancreatic islets and lymph nodes and analyzed by flow cytometry. (A) FACS analysis in the pancreatic islets. Upper panel: proportion of CD4^+^ and CD8^+^ T cells detected within the islets; lower panel: tet/p79 tetramer staining (MHC class II tetramer carrying a BDC2.5 T cell-specific peptide p79) in the CD4^+^ T cell gate (p<0.04). Histogram: islet infiltrating CD4^+^ and CD8^+^ T cell count (*p<0.003). (B) FACS analysis in the pancreatic lymph nodes (PLN). Upper panel: distribution of CD4^+^ and CD8^+^ T cells, lower panel: tet/p79 tetramer staining in the CD4^+^ T cell gate. (C) Proliferation of BDC2.5 T cells in the pancreatic lymph nodes (PLN) and islets of recipient NOD mice measured by CFSE staining in the CD4^+^Tet/p79^+^ T cell gate. The results shown here are representative of three independent experiments.

The diabetogenic CD4^+^ Th1 cell clone BDC2.5, from which the transgenic TCR was isolated, is specific for a still non characterized beta-cell-specific autoantigen presented in the context of NOD (H2^g7^) class II MHC molecules [32]–[34]. Transgenic BDC2.5 cells are highly pathogenic. When the transgene is expressed in the NOD-SCID or RAG^−/−^ NOD backgrounds, all T cells express the BDC2.5 TCR (since recombination of endogenous TCRα chains is prevented) resulting in a massive destructive infiltration of pancreatic islets (insulitis) and overt diabetes two to three weeks after birth [29], [33], [34]. As described by the group of D. Mathis, when adoptively transferred into intact NOD mice, transgenic BDC2.5 T cells rapidly migrate to the pancreatic lymph nodes where they actively proliferate before invading the islets [35]. We used this model to better assess the effect of anti-GITR on effector T cells in vivo. CFSE-labeled transgenic CD4^+^BDC2.5 T cells were injected i.v. into 3-week-old NOD mice and their distribution and expansion was monitored in anti-GITR-treated and control recipients. Anti-GITR antibody was administered on day 0, 1 and 4 following BDC2.5 cell infusion; pancreatic lymph nodes as well as pancreatic islets were recovered on day 7. To specifically detect CD4^+^BDC2.5 T cells, we used I-A^g7^ MHC class II tetramers (tetp79) presenting a synthetic peptide 1040-79 (p79) selectively recognized by the BDC2.5 clone [30], [36]. The number of both CD4^+^ and CD8^+^ T cells accumulating within the islets was 3–4 fold higher in recipients treated with anti-GITR antibody as compared to controls (figure 4A, p<0.003). Interestingly, in the islet infiltrate, the proportion of CD8^+^ T cells increased from 18% to 31% while, in parallel, in pancreatic lymph nodes it decreased from 32% to 23% (figure 4B). The proportion of total CD4^+^ T cells remained unchanged in these two compartments (figure 4A and B). However, a significantly higher proportion of BDC2.5 T cells (stained by the Ag7/p79 tetramer) was observed in the islets, but not in pancreatic lymph nodes, of recipients treated with anti-GITR antibody as compared to controls (11.5% versus 1.3%, figure 4A, p<0.04). BDC2.5 T cells actively proliferated in pancreatic lymph nodes and in the islets of recipient mice. This proliferation was clearly enhanced following anti-GITR treatment; 84% of islet-infiltrating BDC2.5 T cells underwent more than 5 divisions (figure 4C). Similarly, only 21% of BDC2.5 T cells present in pancreatic lymph nodes of anti-GITR-treated animals were non-dividing as compared to 49% in the control mice. BDC2.5 T cells were mostly found in the close vicinity of the target organ. A minor proportion of them were detected in the spleen and the mesenteric lymph nodes of recipient mice; they did not proliferate and their frequency did not increase following anti-GITR treatment (data not shown).

### 2) GITR Ligation Does Not Abrogate Treg Function

#### a) No effect of *in vivo* and *in vitro* anti-GITR treatment on Treg function

**Figure 5:**
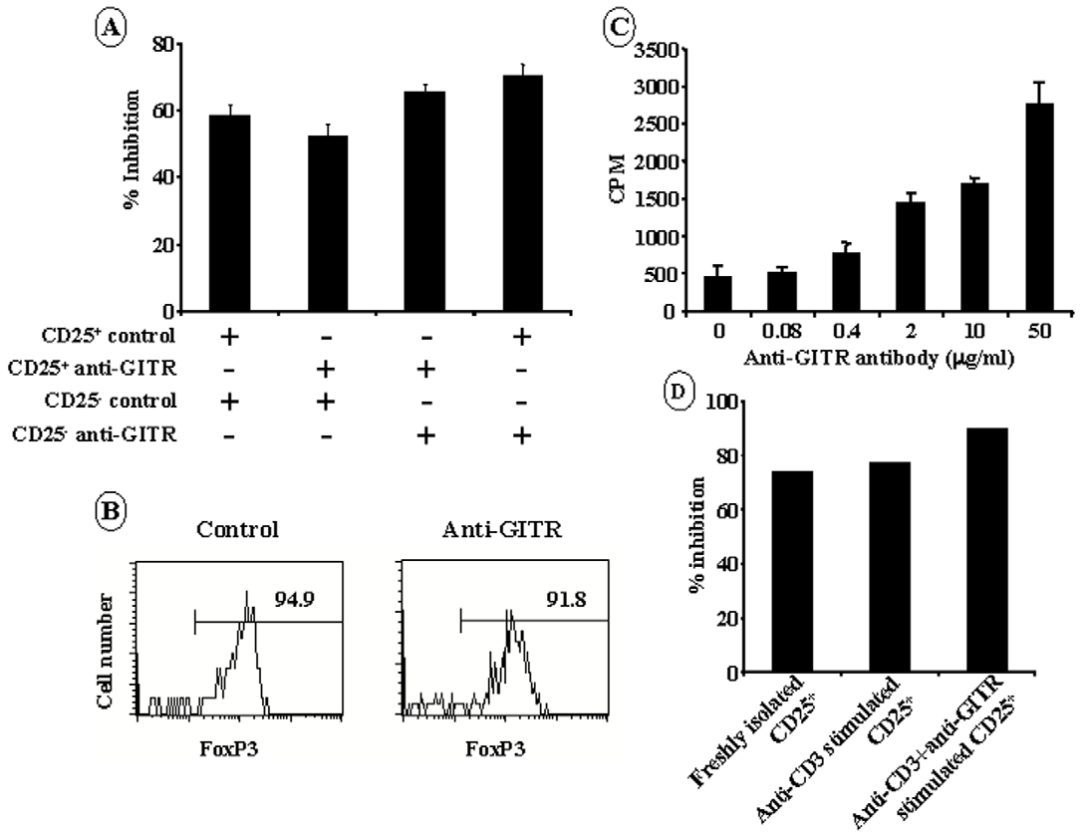
GITR triggering does not abrogate regulatory T cell function. (A) *In vitro* suppressive capacities of CD4^+^CD25^+^ T cells recovered from anti-GITR antibody-treated NOD mice. CD4^+^CD25^−^ and CD4^+^CD25^+^ T cells were recovered from the spleen cell of 6-week-old NOD mice injected with anti-GITR antibody (0.8 mg on day 10, 17 and 24) or of control mice. Criss-cross co-cultures were performed by incubating the CD4^+^CD25^+^ T cells from anti-GITR-treated NOD mice with CD4^+^CD25^−^ T cells from control animals and inversely. The two T cell subsets (CD25^+^ and CD25^−^) were cultured at a 1/1 ratio in presence of APCs and CD3 antibody for 72 hrs. Data represent the mean of 4 experiments. (B) FoxP3 expression by CD4^+^CD25^+^ T cells recovered from the spleen of 5-week-old NOD mice treated with anti-GITR antibody. (C) CD4^+^CD25^+^ T cells from unmanipulated NOD mouse spleen were stimulated with coated CD3 antibody (0.5 µg/ml) in the presence of increasing concentrations of anti-GITR antibody ranging from 0.08 to 50 µg/ml. (D) CD4^+^CD25^+^ T cells were recovered from 6-week-old NOD mice and cultured with CD3 antibody (0.5 µg/ml) or CD3+GITR antibodies (50 µg/ml) for 48 hrs. Cells were harvested, washed and their suppressive capacity was evaluated after 72 hrs incubation with freshly isolated CD4^+^CD25^−^ T cells. Freshly isolated CD4^+^CD25^+^ T cells were used as controls. The results shown here are representative of at least three experiments.

It has been suggested that ligation of GITR inhibits the suppressive capacity of Tregs [1], [4], [8], [37]. We investigated the *in vitro* suppressive capacities of the CD4^+^CD25^+^ T cells isolated from the spleen of GITR antibody-treated mice using the conventional non antigen-specific co-culture model. CD4^+^CD25^+^ and CD4^+^CD25^−^ T cells were recovered from NOD mice 2 weeks after the last anti-GITR injection. As shown on figure 5A, CD4^+^CD25^+^ T cells from anti-GITR-treated NOD mice efficiently suppressed the proliferation of CD4^+^CD25^−^ T cells recovered from the same donor. To further examine if GITR triggering altered the regulatory properties of the CD4^+^CD25^+^ subset, we performed criss-cross co-cultures. CD4^+^CD25^+^ T cells from anti-GITR-treated NOD mice suppressed proliferation of CD4^+^CD25^−^ T cells from control mice as efficiently as Tregs from the same control mice (figure 5A). FoxP3 expression was investigated and was detected in the majority of the CD4^+^CD25^+^ T cells both in anti-GITR-treated and in control animals (figure 5B).

Using another *in vitro* experimental design, we studied the effect of GITR triggering on TCR-stimulated CD4^+^CD25^+^ T cells recovered from non-manipulated NOD mice. We showed that addition of increasing concentrations of the anti-GITR agonist antibody resulted in a dose-dependent enhancement of CD4^+^CD25^+^ T cell proliferation (figure 5C) i.e. CD4^+^CD25^+^ T cells lost their anergic state in response to CD3 antibody. However, these CD4^+^CD25^+^ T cells that have been previously stimulated with a combination of CD3 and GITR antibodies exhibited suppressive capacities similar to the one afforded by freshly isolated CD4^+^CD25^+^ T cells or CD4^+^CD25^+^ T cells cultured with CD3 antibody alone (figure 5D).

#### b) The CD28^−/−^ NOD mouse model

**Figure 6:**
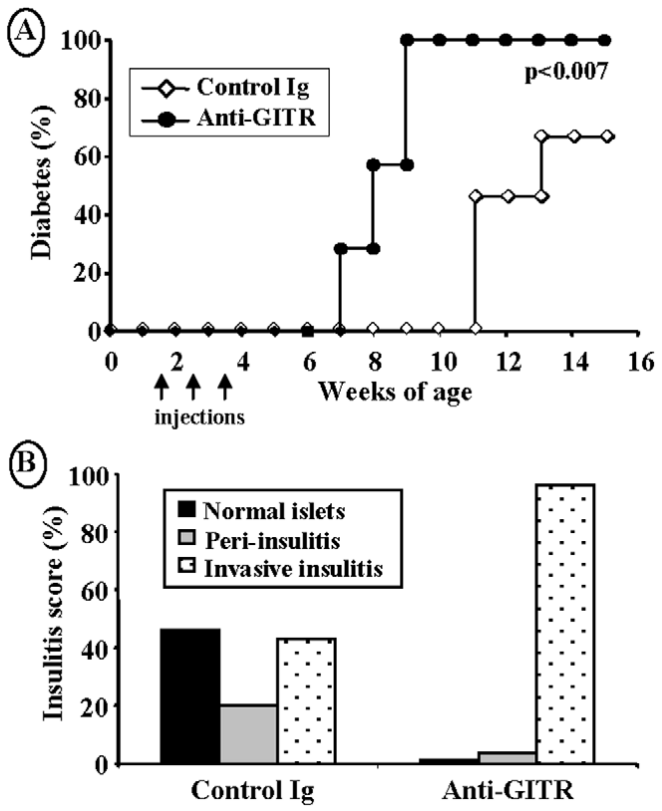
Acceleration of diabetes incidence in CD28^−/−^ NOD mice following administration of anti-GITR antibody. (A) Ten day-old female CD28^−/−^ NOD mice (n = 8) were treated with anti-GITR antibody or purified Igs. The dose used was 0.8 mg/injection/mouse i.p. once a week on d10, d17 and d24 of life. Anti-GITR antibody treatment significantly exacerbated diabetes onset in all animals used (p<0.007). (B) Histological analysis of pancreas recovered from 9-week-old female CD28^−/−^ NOD mice injected with anti-GITR antibody or control Igs. Invasive insulitis was clearly worsened in anti-GITR-treated animals (p<0.011).

To further confirm that anti-GITR treatment selectively acted on effector T cells but spared Tregs, we used NOD mice deficient in the *cd28* encoding gene (CD28^−/−^ NOD). These mice are deprived of natural CD25^+^FoxP3^+^ natural Tregs and they exhibit exacerbated Th1 responses and accelerated diabetes [38], [39]. Anti-GITR antibody treatment was performed in 10 day-old female CD28^−/−^ NOD mice (0.8 mg/injection on d10, d17 and d24 of life). As expected, 66.7% of control animals that received control IgGs showed overt disease by 15 weeks of age (figure 6A). Anti-GITR-treated mice showed acceleration of disease onset reaching 100% of diabetes incidence by 9 weeks of age. Histological examination of pancreata recovered from 9-week-old anti-GITR antibody-treated CD28^−/−^ NOD mice showed more than 95% of massively infiltrated and destroyed islets (as compared to 43% in the control group) (figure 6B).

### 3) Blockade of GITR Ligand Is Effective at Preventing Disease Progression

#### a) Treatment of NOD mice with a short anti-GITRL course protects from diabetes

As a whole, the data presented above clearly suggest that GITR triggering promotes exacerbation of autoimmune diabetes consequent to the activation of diabetogenic T cells without significantly affecting the Treg pool. We therefore hypothesized that blocking GITR/GITRL interaction may represent a promising therapeutic strategy to control diabetes development.

**Figure 7:**
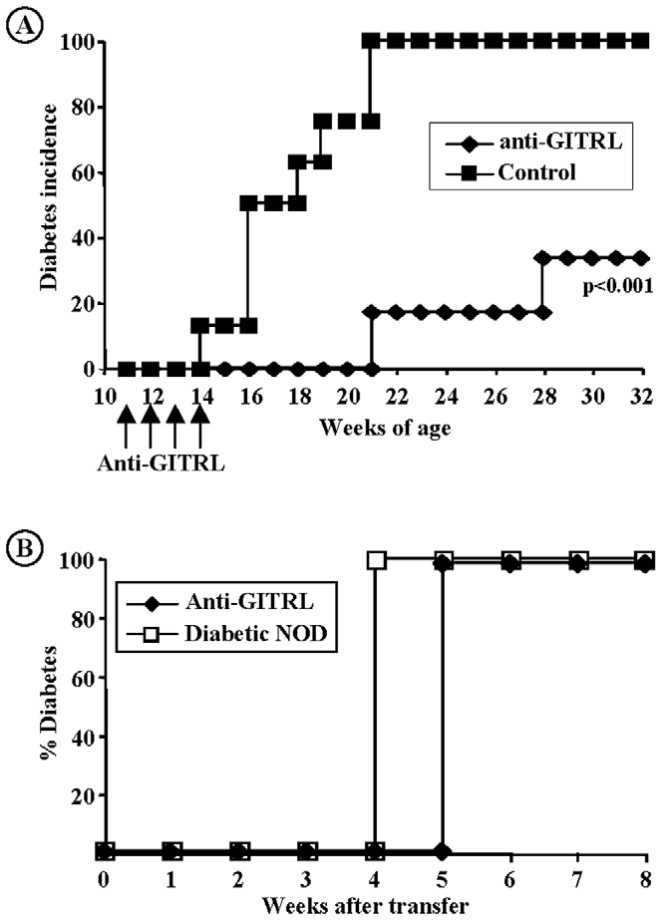
*In vivo* administration of anti-GITRLigand antibody protects from diabetes development. (A) Treatment with anti-GITRL antibody started at 11 weeks of age in pre-diabetic NOD mice, 1 mg antibody/week i.p. on week 11 to 14 (n = 8). Significant protection was observed as compared to control animals (33.3% diabetes versus 100% in the control group at 32 weeks of age, p<0.001). (B) Adoptive transfer into NOD-SCID recipients of CD25^−^CD62L^−^ T cells recovered either from the spleen of 32-week-old diabetes-free anti-GITRL antibody-treated NOD mice (10^6^/mouse) or from the spleen of overtly diabetic animals. Both populations were able to transfer diabetes with a similar efficiency.

To directly test this hypothesis, a monoclonal anti-GITRL antibody (clone YGL 386.2), that interferes with the GITR/GITRL interaction, was injected intraperitonealy (1 mg/injection/week) at a late pre-diabetic stage (11-week-old) for only 4 consecutive weeks. Diabetes incidence was significantly reduced: 0% versus 78% diabetes in the control group at 20 weeks of age, 16.6% versus 100% at 25 weeks of age and 33% versus 100% diabetes at 30 weeks of age (figure 7A). Phenotypic analysis of various lymphoid organs (spleen, mesenteric, pancreatic lymph nodes) did not reveal any major modification of the T cell compartment as compared to untreated NOD mice (data not shown). In particular, the proportion and the suppressive functions of CD4^+^CD25^+^FoxP3^+^ regulatory T cells recovered from anti-GITRL antibody-treated NOD mice at different time points following treatment were not modified as compared to age-matched controls (data not shown).

#### b) Anti-GITRL antibody-protected NOD mice harbor pathogenic T cells

We next tested whether protected NOD mice still harbored pathogenic T cells. Total splenocytes were harvested from 32-week-old anti-GITRL antibody-treated diabetes-free NOD mice and were depleted of regulatory T cells i.e. CD4^+^CD25^+^ T cells and CD4^+^CD62L^+^ T cells [39]–[41]. The CD25^−^CD62L^−^ T cell fraction, that included diabetogenic effectors [42], was adoptively transferred into 6-week-old NOD-SCID mice (10^6^/recipient). As shown on figure 7B, CD25^−^CD62L^−^ T cells from anti-GITRL-treated donors were still able to transfer disease as efficiently as T cells from overtly diabetic NOD mice. This result argues against the deletion of diabetogenic T cells following the anti-GITRL antibody treatment.

### 4) Blockade of the GITR/GITRL Pathway Reduced *In Vivo* Proliferation and Migration of Effector T Cells to the Pancreas

The data presented above indirectly suggest a critical role for the GITR/GITRL interaction in the migration of pathogenic effector T cells to the target tissue. To assess more directly whether blockade of the GITR/GITRL pathway directly interfered with the activation and/or migration of effector T cell reactivity, we took advantage of the experimental design described above using CFSE-labeled CD4^+^BDC2.5 T cells. BDC2.5 T cells were infused into 4-week-old NOD mice that were then treated with 1 mg anti-GITRL antibody on day 0, 1 and day 4. Spleen, mesenteric and pancreatic lymph nodes as well as pancreatic islets were recovered on day 7.

**Figure 8:**
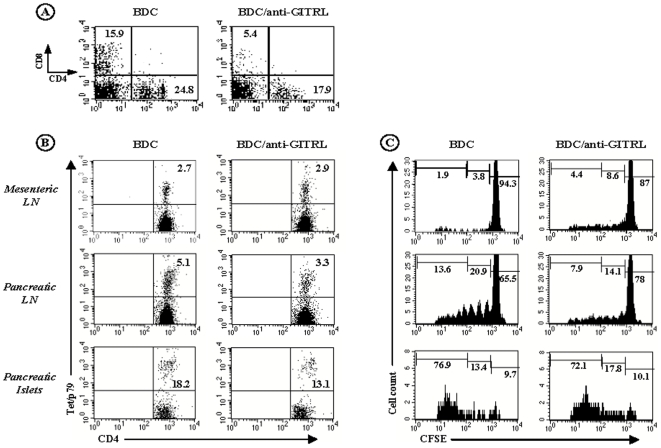
Blockade of GITR/GITRLigand pathway decreased BDC2.5 T cell migration and proliferation in the pancreatic lymph nodes. Three to four-week-old NOD mice were infused with 10^7^ CFSE-labeled CD4^+^BDC2.5 T cells and were treated or not with anti-GITRL antibody on day 0, 1 and day 4 after transfer. Recipient mice were sacrified on day 7 and cell suspensions were harvested from mesenteric and pancreatic lymph nodes and from pancreatic islets. (A) Staining of islet-infiltrating CD4^+^ and CD8^+^ T cells in recipient NOD mice. Less T cells were detected after anti-GITRL treatment (p<0.05 for CD4^+^ T cells, p<0.02 for CD8^+^ T cells). (B) Detection of BDC2.5 T cells using the Tet/p79 tetramer in various organs after gating on CD4^+^ T cells. Reduced numbers of BDC2.5 T cells were found in pancreatic lymph nodes and islets of anti-GITR-treated recipients (p<0.05 and p<0.04, respectively). (C) Proliferation of the infused BDC2.5 T cells followed by the CFSE staining in the CD4^+^Tet/p79^+^ T cell gate. BDC2.5 T cells were present in reduced number and proliferated less the pancreatic lymph nodes of anti-GITRL antibody-treated NOD mice. The results shown here are representative of three independent experiments.

The analysis of immune cells infiltrating the pancreatic islets showed that less CD4^+^ T cells were detected after injection of anti-GITRL antibody (17.9% versus 24.8% in islets from untreated animals) (figure 8A, p<0.05). Among these CD4^+^ T cells, 13.1% were stained with tetp79 as compared to 18.2% in controls (figure 8B, p<0.04). In other words, proliferating BDC2.5 T cells represent 2.3% of the total lymphocytes found within the pancreatic islets of anti-GITRL-treated recipient mice as compared to 4.5% in untreated animals (data not shown). Interestingly, the proportion of CD8^+^ T cells present within the islets was decreased after anti-GITRL antibody treatment as compared to untreated recipient NOD mice (figure 8A, p<0.02).

**Figure 9:**
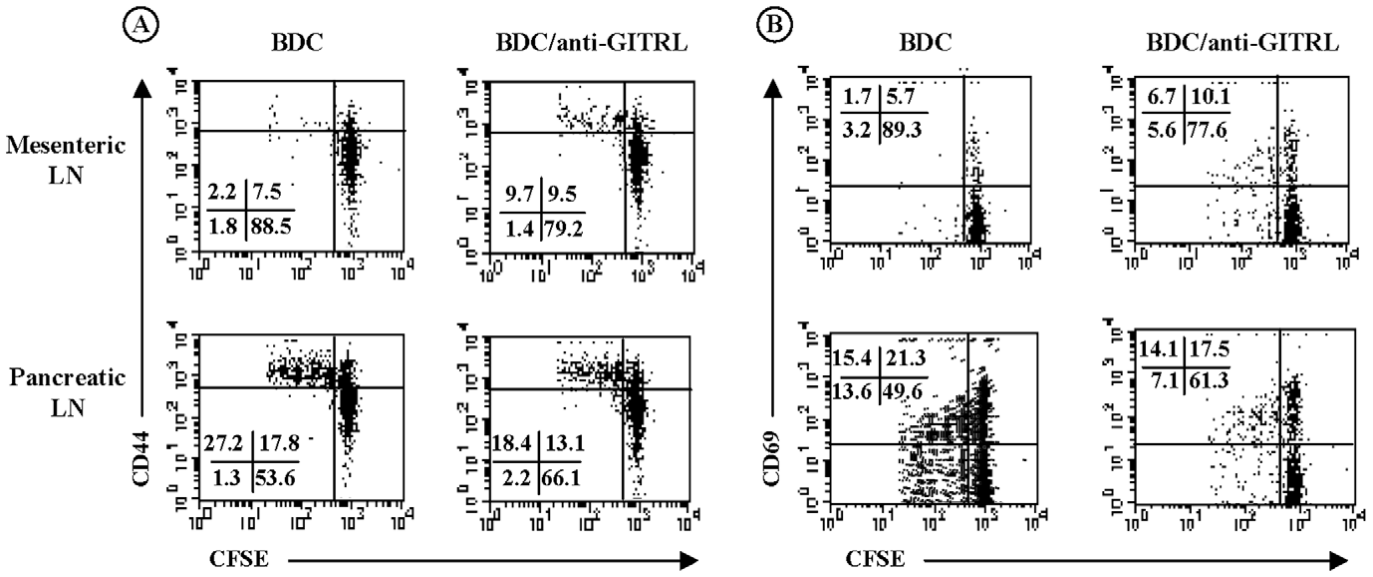
Blockade of GITR/GITRLigand pathway decreased BDC2.5 T cell activation in the pancreatic lymph nodes. CD44 (panel A) and CD69 (panel B) staining were performed on CFSE-labeled BDC2.5 T cells that were previously infused into 3 to 4-week-old NOD mice. Recipient mice were treated or not with anti-GITRL antibody on day 0, 1 and 4 after cell infusion. Mesenteric and pancreatic lymph nodes were recovered on day 7. The proportion of dividing cells (CFSE^low^) expressing CD44^high^ and CD69 decreased in the pancreatic lymph nodes of anti-GITRL antibody-treated NOD mice (p<0.05 and p<0.04, respectively). BDC2.5 T cells that migrated to mesenteric lymph nodes showed increased proliferation as well as CD44^high^ and CD69 expression.

BDC2.5 T cells accumulated less (3.3% of total CD4^+^ T cells versus 5.1% in untreated animals, figure 8B, p<0.05) and, importantly, proliferated less in pancreatic lymph nodes of NOD mice treated with anti-GITRL antibody (Figure 8C). Only 22% of the BDC2.5 T cells underwent at least one division after administration of anti-GITRL antibody as compared to 34.5% without treatment (figure 8C). Phenotypic analysis was performed on the BDC2.5 T cells infused into NOD mice treated or not with anti-GITRL antibody. Expression of CD44^high^ was clearly detected on T cells that proliferated in the pancreatic lymph nodes of recipient mice (figure 9A). However, correlating with the previous results (figure 8), less BDC2.5 T cells were CFSE^low^CD44^high^ after administration of anti-GITRL antibody (figure 9A, p<0.05). Similarly, BDC2.5 T cells that migrated to the pancreatic lymph nodes of anti-GITRL antibody-treated mice expressed lower levels of CD69 (figure 9B). Interestingly, we observed that CD69 staining intensity decreased as cells divided. In control animals, BDC2.5 T cells (13.6%) that underwent several rounds of cell division did not express CD69 (figure 9B). This CFSE^low^CD69^−^ population is present at a lower proportion in anti-GITRL antibody-treated mice (p<0.04). CD69 is an early activation marker. The lack of expression of CD69 on a fraction of proliferating CFSE^low^ T cells, as well as their high expression of CD44, suggest that, in untreated NOD mice, BDC2.5 T cells have already gone through several rounds of activation and exhibit an effector/memory-like phenotype. This is not the case in anti-GITRL antibody-treated mice.

Some BDC2.5 T cells did proliferate and acquired the activation markers CD44^high^ and CD69 in the mesenteric lymph nodes of anti-GITRL antibody-treated mice (figure 9A and B). In contrast, BDC2.5 T cells detected in the spleen of recipient NOD mice (around 3% of total T cells) did not proliferate nor express CD44^high^ or CD69, independently of any antibody treatment (data not shown).

### 5) The Protective Effect of Anti-GITRL Is Independent of the Presence of Treg

**Figure 10:**
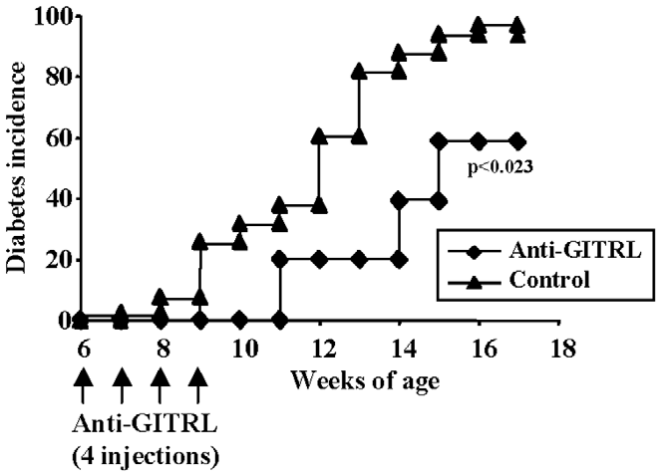
Anti-GITRLigand antibody treatment delays diabetes in CD28^−/−^ NOD mice. (A) Anti-GITRL antibody was administered into pre-diabetic 6-week-old CD28^−/−^ NOD mice, 1 mg/injection/week for 4 consecutive weeks (n = 8). Diabetes development was significantly delayed as compared to the control group (p<0.023).

To further investigate the effect of blocking GITR/GITRL pathway on effector T cell functions, pre-diabetic 6-week-old CD28^−/−^ NOD mice were treated with an anti-GITRL antibody (1 mg/injection on week 6 to 9). Administration of this anti-GITRL antibody significantly delayed (but did not abrogate) diabetes development in CD28^−/−^ NOD mice (figure 10), as opposed to what observed with the agonist anti-GITR antibody (figure 6A).

## Discussion

Type 1 diabetes is a prototypic organ-specific autoimmune disease resulting from the selective destruction of insulin-secreting beta-cells within pancreatic islets of Langerhans by an immune-mediated inflammation involving autoreactive CD4^+^ and CD8^+^ T lymphocytes and monocytic cells which infiltrate pancreatic islets (insulitis) [43]. Current treatment is substitutive i.e. chronic use of exogenous insulin which is, in spite of significant advances, still associated with major constraints (multiple daily injections, risks of hypoglycemia) and a lack of effectiveness over the long term in preventing severe degenerative complications. Finding a cure for autoimmune diabetes is a real health challenge since its incidence steadily increases in industrialized countries [44], [45]. Our present results demonstrate a key role of the GITR/GITRL pathway in the progression of autoimmune diabetes and point to its potential as a therapeutic target. On one hand, GITR triggering following *in vivo* administration of an agonistic anti-GITR monoclonal antibody promotes a clear-cut exacerbation of disease. On the other hand, adequate pharmacological blockade of this pathway following short-term *in vivo* treatment with an anti-GITRL antibody is effective at protecting from disease development. For the sake of clarity, we shall discuss these two aspects of our work consecutively.

The capacity to significantly up-regulate immune responses upon GITR triggering is quite remarkable and covers the whole spectrum of antigens that have been studied, including infectious and tumor antigens, autoantigens and alloantigens. This effect was initially described by the group of S. Sakaguchi in autoimmunity with the DTA-1 antibody (a rat anti-mouse GITR monoclonal) they had produced using natural CD4^+^CD25^+^ Tregs as an immunogen [1]. The DTA-1 antibody blocked the *in vitro* suppressive ability of CD4^+^CD25^+^ T cells. Additional experiments confirmed the agonistic properties of DTA-1 through active GITR signaling and not mere blockade of the receptor; only the intact antibody but not Fab fragments mediated the effect [1]. *In vivo*, only 3 injections of the anti-GITR antibody (once a week for 3 weeks) to very young BALB/c mice (2 week-old) induced by 3 months of age histological and serological evidence of autoimmune gastritis [1], a situation partly resembling that induced following elimination of CD4^+^CD25^+^ T cells by day 3 thymectomy in BALB/c mice [46]. In EAE, the same anti-GITR antibody significantly increased disease severity and CNS inflammation and induced elevated levels of antigen-specific T cell proliferation and cytokine production [23]. Similarly, both Th1- and Th2-mediated inflammatory disorders (collagen-induced arthritis and asthma, respectively) were exacerbated after injection of anti-GITR [24]. In the present report, we extended these data to a spontaneous autoimmune insulin-dependent diabetes model, the NOD mouse, that recapitulates many aspects of the human disease. Using another agonistic anti-GITR antibody, 2F8, we found a very significant acceleration of disease onset following a short treatment course (3 injections, once a week) started in young mice (10 days of age). As in the case of DTA-1, our 2F8 antibody did not eliminate GITR-expressing cells. Histological autoimmune sialitis, that is spontaneously observed in NOD mice, was also worsened in anti-GITR-treated animals. However, in contrast to what was observed in the BALB/c mouse strain [1], severe gastritis was not induced in NOD mice. In the autoimmune or inflammatory models cited above, the authors could not conclude whether the disease accelerating effect of GITR triggering was due to a decrease in the functional capacity of Tregs or to a stimulating/costimulatory effect on pathogenic T cells that also express GITR. We addressed this important issue in the NOD mouse model. Of note are the higher proportions of CD4^+^ and CD8^+^ T cells showing an activated phenotype in anti-GITR-treated mice as compared to controls pointing, though indirectly, to an accelerated maturation/differentiation of peripheral T cells. Moreover, we collected evidence arguing for a major costimulatory effect on pathogenic/diabetogenic T cells of anti-GITR antibody contrasting with a lack of effect on Tregs.

First, *in vitro*, increasing concentrations of anti-GITR antibody exerted a costimulatory effect on the proliferative capacity and/or the IFN-γ production of CD4^+^CD25^−^ T cells (depleted of Tregs) and CD8^+^ T cells in response to a polyclonal stimulation (i.e. anti-CD3). The same effect was observed in the context of an autoantigen-specific stimulation using CD4^+^ T cells from BDC2.5 NOD mice (that express an islet autoantigen-specific transgenic T cell receptor): a dose-dependent costimulatory effect was observed upon addition of anti-GITR as assessed by the IFN-γ production. Similarly, CD4^+^CD25^−^ T cells recovered *ex vivo* from anti-GITR-treated mice exhibited a significantly enhanced proliferative capacity to suboptimal doses of anti-CD3. These findings fit with observations reported in models of EAE, asthma and collagen-induced arthritis showing enhanced CD4^+^ T cell activation, proliferation and cytokine production after administration of agonist anti-GITR antibodies [23], [24].

Secondly, using an *in vivo* model in which CFSE-labeled pathogenic BDC2.5 NOD CD4^+^ T cells were transferred into young syngeneic recipients, we showed that, in anti-GITR-treated recipients, the migration of pathogenic effectors to the target tissue and its draining lymph nodes (i.e. within the infiltrated islets and pancreatic lymph nodes) as well as their *in situ* proliferation was greatly enhanced as compared to untreated control recipients. This phenomenon was accompanied by an enhanced CD8^+^ T cell migration within the islets of treated animals that may result from the combined effect of GITR triggering and the activating/chemoattracting factors produced by activated CD4^+^BDC2.5 T cells.

Third, no effect of anti-GITR antibody on Treg function was found as assessed by a completely normal ability to suppress, in the conventional coculture system, of Tregs pre-treated *in vitro* with anti-GITR or recovered *ex vivo* from anti-GITR-treated NOD mice. These results confirm and extend previous results by McHugh et al. showing that GITR triggering on Tregs during a pre-activation phase (with anti-CD3 and IL-2) did not abrogate their suppressive capacity in a subsequent coculture assay with anti-CD3 or peptide-specific stimulated CD4^+^CD25^−^ T cells [4]. In addition, *in vivo*, we were not able to detect in any lymphoid organ a significant increase in the number or proportion of Tregs at any time-point after administration of anti-GITR antibody although *in vitro* addition of anti-GITR increased Treg proliferation in response to CD3-specific antibody stimulation.

This lack of effect on regulatory CD4^+^CD25^+^ T cells was reinforced by the fact that the disease accelerating effect of anti-GITR treatment could be obtained in the absence of thymus-derived natural Tregs that is the case of NOD mice deficient for CD28 (CD28^−/−^ NOD). These findings contrast with initial *in vitro* reports suggesting that ligation of GITR blocked the suppressive ability of freshly isolated Tregs [1], [4]. This apparent contradiction may be reinterpreted in the light of more recent results by Stephens et al., using GITR^−/−^ mice, demonstrating that it is the engagement of GITR on effector T cells (but not on Tregs) which provides a co-activating signal rendering these cells resistant to regulation and as a consequence abrogating suppression [6]. Thus, in the autoimmune setting, GITR may influence the sensitivity of autoreactive T cells to stimulation by the cognate autoantigens, leading to an increased autoreactivity not properly controlled by Tregs. This possibility is further supported by data showing that GITR signaling lowers the threshold of T cell activation [47]. As a whole, our data suggest that the activation, proliferation and migration of effector T cells to the target tissue constitute the primary costimulatory effect of GITR triggering *in vivo*, overcoming the potential impact of anti-GITR on Tregs.

Now, coming to blockade of GITR signaling, the salient result is that it effectively protects from disease development. The original and interesting finding here is that significant protection was achieved even when anti-GITRL antibody treatment started at 11 weeks of age i.e. a late pre-diabetic stage characterized by an already severe islet inflammation. Experiments are ongoing using higher cumulated dosages of anti-GITRL antibody to improve the effect (i.e. complete protection from diabetes development). In addition, work is also in progress to test if GITRL antibody therapy may also be effective at reversing recent onset established diabetes in NOD mice.

Interestingly, anti-GITRL antibody treatment applied to the two *in vivo* models used to study effector T cells (i.e. CD28^−/−^ NOD mice and adoptive transfer of CFSE-labeled BDC2.5 T cells) gave opposite results as compared to anti-GITR agonist antibody treatment. Diabetes was significantly delayed yet not abolished in anti-GITRL antibody-treated CD28^−/−^ NOD mice and BDC2.5 T cells showed reduced migration and proliferation in the islets and pancreatic lymph nodes of anti-GITRL-treated recipient NOD mice as compared to untreated animals. As a mirror image with what found in the islets of anti-GITR-treated recipients, this reduced migration of CD4^+^ diabetogenic cells in anti-GITRL antibody-treated mice correlated with a quite dramatic reduction in the proportion of infiltrating CD8^+^ T cells. Altogether, these results highlight that changes in antigen-specific CD4^+^ T cell migration and proliferation appear to significantly impact the recruitment of CD8^+^ effectors to the target tissue.

Two possibilities can explain that, although delayed, diabetes still occurred in anti-GITRL antibody-treated CD28^−/−^ NOD mice. First, due to the absence of naturally occurring CD4^+^CD25^+^ Tregs, these mice exhibited accelerated disease development compared to wild-type NOD mice (overt diabetes is usually observed by 8 weeks of age). Administration of anti-GITRL antibody was performed at 6 weeks of age once too many islets were already aggressively infiltrated to achieve significant arrest of the process. Secondly, one cannot at this point formally exclude a role for CD4^+^CD25^+^ Tregs in the protective effect of anti-GITRL antibody. It is true that we did not observe any major modification in terms of number/proportions or suppressive functions in the various lymphoid organs (spleen, mesenteric and pancreatic lymph nodes) recovered from protected wild-type NOD mice. However, these Tregs may be important for controlling pathogenic T cells that did not migrate into pancreatic islets and are still present at the periphery as shown by the efficient transfer of diabetes into NOD-SCID recipients by splenic T cells from diabetes-free anti-GITRL-treated NOD mice.

As a whole, these results suggest that disease protection obtained following blockade of the GITR/GITRL pathway may rely on two distinct but not mutually exclusive mechanisms that are first, a limitation of the activation and migration of effector cells to the target tissue and, second, the maintenance of peripheral self-tolerance via CD4^+^CD25^+^ T cell-mediated regulation.

It will be important to study the GITRL expression and its regulation on APCs during disease progression in NOD mice. In keeping with data reported by Kamimura et al. in a model of contact hypersensitivity [48], one may hypothesize that APCs expressing GITRL (that may increase in the pro-inflammatory environment created by the insulitis within pancreatic islets) enhance the homing potential of GITR-expressing autoreactive T cells.

From a more practical, clinically oriented perspective, these results point to the use of anti-GITRL as a novel avenue for the treatment of autoimmune type 1 diabetes. The incidence of autoimmune diabetes has tremendously increased in industrialized countries over the last three decades [49] and predictions for future years are alarming [44], [45]. According to two recent reports, disease incidence has accelerated since 2000 and if present trends continue, the number of new cases diagnosed at or before 14 years of age will double in the next 15 years with onset at younger age (0–4 years) [44], [45]. Therefore, establishing safe immune intervention strategies, that may provide a real cure for the disease, is both an urgent need and a real medical health challenge. Given the young age of the affected population, any candidate therapy must be safe and avoid a sustained depression of immune responses thus aiming at inducing or, in the case of established diabetes, restoring immune tolerance to target autoantigens. Major progress towards that aim has been made over the last 20 years and promising strategies are presently studied both to reverse established disease or to prevent it based on the use of candidate autoantigens or immune modulating drugs [50]–[53]. Although very encouraging, results so far are not totally satisfactory, and, as in many other clinical situations, one may predict that major improvements will be achieved as dosing schedules are adjusted and drug combinations are performed.

Our present data point to anti-GITRL antibodies as very good potential candidates whose capacity to block the migration to the target organ and the activation of pathogenic effectors appears fully complementary to that of the other agents presently tested which preferentially target T-cell mediated regulatory pathways [51], [53]–[59]. The question of whether an autoantigen-specific effect may be obtained is still open. One may speculate that although GITR is expressed on all T lymphocytes, blockade of GITR/GITRL pathway may promote differential signaling depending on the nature and the functional capacity of T cell targets (i.e. a naïve T cell, an activated or a memory T cell, a Treg), thus determining the ‘quality’ of the GITR antibody-mediated signal and the resulting outcome.
